# Intervention protocol: OPtimising thERapy to prevent avoidable hospital Admission in the Multi-morbid elderly (OPERAM): a structured medication review with support of a computerised decision support system

**DOI:** 10.1186/s12913-020-5056-3

**Published:** 2020-03-17

**Authors:** Erin K. Crowley, Bastiaan T. G. M. Sallevelt, Corlina J. A. Huibers, Kevin D. Murphy, Marco Spruit, Zhengru Shen, Benoît Boland, Anne Spinewine, Olivia Dalleur, Elisavet Moutzouri, Axel Löwe, Martin Feller, Nathalie Schwab, Luise Adam, Ingeborg Wilting, Wilma Knol, Nicolas Rodondi, Stephen Byrne, Denis O’Mahony

**Affiliations:** 1 Pharmaceutical Care Research Group. School of Pharmacy, Cavanagh Pharmacy Building, University College Cork, Cork, Ireland; 2 Department of Clinical Pharmacy, Division Laboratory and Pharmacy, University Medical Center Utrecht, Utrecht University, Utrecht, The Netherlands; 3 Department of Geriatric Medicine and Expertise Centre Pharmacotherapy in Old Persons, University Medical Center Utrecht, Utrecht University, Utrecht, The Netherlands; 4 Department of Information and Computing Sciences, Utrecht University, Princetonplein 5, 3584CC Utrecht, The Netherlands; 5 Department of Geriatric Medicine, Cliniques universitaires Saint-Luc, Université catholique de Louvain UCLouvain, Brussels, Belgium; 6 Institute of Health and Society, Université catholique de Louvain UCLouvain, Brussels, Belgium; 7 Clinical Pharmacy Research Group, Louvain Drug Research Institute, Université catholique de Louvain UCLouvain, Brussels, Belgium; 8 Pharmacy Department, CHU Dinant-Godinne UCL Namur, Université catholique de Louvain UCLouvain, Yvoir, Belgium; 9 Pharmacy Department, Cliniques universitaires Saint-Luc, Université catholique de Louvain UCLouvain, Brussels, Belgium; 10 Department of General Internal Medicine, Inselspital, Bern University Hospital, University of Bern, Bern, Switzerland; 11 Institute of Primary Health Care (BIHAM), University of Bern, Bern, Switzerland; 12 Department of Medicine, University College Cork, Cork, Ireland; 13 Department of Geriatric Medicine, Cork University Hospital, Cork, Ireland

**Keywords:** Geriatric patient, Cluster randomised controlled trial, STOPP/START, Inappropriate prescribing

## Abstract

**Background:**

Several approaches to medication optimisation by identifying drug-related problems in older people have been described. Although some interventions have shown reductions in drug-related problems (DRPs), evidence supporting the effectiveness of medication reviews on clinical and economic outcomes is lacking. Application of the STOPP/START (version 2) explicit screening tool for inappropriate prescribing has decreased inappropriate prescribing and significantly reduced adverse drug reactions (ADRs) and associated healthcare costs in older patients with multi-morbidity and polypharmacy. Therefore, application of STOPP/START criteria during a medication review is likely to be beneficial.

Incorporation of explicit screening tools into clinical decision support systems (CDSS) has gained traction as a means to improve both quality and efficiency in the rather time-consuming medication review process. Although CDSS can generate more potential inappropriate medication recommendations, some of these have been shown to be less clinically relevant, resulting in alert fatigue. Moreover, explicit tools such as STOPP/START do not cover all relevant DRPs on an individual patient level. The OPERAM study aims to assess the impact of a structured drug review on the quality of pharmacotherapy in older people with multi-morbidity and polypharmacy. The aim of this paper is to describe the structured, multi-component intervention of the OPERAM trial and compare it with the approach in the comparator arm.

**Method:**

This paper describes a multi-component intervention, integrating interventions that have demonstrated effectiveness in defining DRPs. The intervention involves a structured history-taking of medication (SHiM), a medication review according to the systemic tool to reduce inappropriate prescribing (STRIP) method, assisted by a clinical decision support system (STRIP Assistant, STRIPA) with integrated STOPP/START criteria (version 2), followed by shared decision-making with both patient and attending physician. The developed method integrates patient input, patient data, involvement from other healthcare professionals and CDSS-assistance into one structured intervention.

**Discussion:**

The clinical and economical effectiveness of this experimental intervention will be evaluated in a cohort of hospitalised, older patients with multi-morbidity and polypharmacy in the multicentre, randomized controlled OPERAM trial (OPtimising thERapy to prevent Avoidable hospital admissions in the Multi-morbid elderly), which will be completed in the last quarter of 2019.

**Trial registration:**

Universal Trial Number: U1111-1181-9400 Clinicaltrials.gov: NCT02986425, Registered 08 December 2016.

FOPH (Swiss national portal): SNCTP000002183. Netherlands Trial Register: NTR6012 (07-10-2016).

## Contributions to the literature

- Explicit prescribing appropriateness criteria such as STOPP/START reduce adverse drug reactions and medication costs in older persons yet implementation in healthcare systems globally is low.
- STRIP is a robust system that has been successfully implemented in Belgium, Ireland, the Netherlands, and Switzerland as part of the OPERAM trial and could be integrated in any healthcare system.

## Background

The global population aged over 65 years is rapidly increasing such that by 2060 approximately one-third of the European population is projected to be over 65 years [1]. In this ageing population, there is a higher prevalence of multi-morbidity, which is in turn associated with greater mortality [2], decreased quality of life (QoL) and increased number of hospital admissions [3]. Moreover, these patients are frequently exposed to multiple medications in the context of their multi-morbidity i.e. multiple chronic diseases usually engender multiple prescriptions, also known as polypharmacy. Although polypharmacy has several definitions, the most broadly accepted is that of the concurrent use of ≥5 medications [4]. Polypharmacy in older patients has been repeatedly shown to result in negative consequences such as increased healthcare costs, adverse drug reactions (ADRs), adverse drug-drug interactions (DDI) and drug-related hospital admissions [5–7]. Importantly, the risk of either ADR or DDI occurrence increases with the number of medications prescribed [8, 9]. Despite this, a recent study demonstrated that across specific European countries, the issue of problematic polypharmacy has not been widely addressed [10].

Several different approaches to optimise prescription medication in older people have been reported [11, 12]. In spite of a general lack of evidence for their significant impact on health-related outcomes, a Cochrane review did find that one particular approach was beneficial in reducing inappropriate polypharmacy [13], i.e. the novel geriatric-specific inappropriate prescribing criteria called Screening Tool of Older Persons’ Prescriptions (STOPP) and Screening Tool to Alert to Right Treatment (START) [14]. The first of a series of 5 randomised controlled trials (RCTs) using the STOPP/START criteria as an intervention demonstrated that the use of these criteria significantly improved prescribing appropriateness up to 6 months after discharge in a cohort of older, hospitalised patients [9]. Further refinements to the criteria resulted in the publication of STOPP/START version 2 [15] and subsequent studies have shown that application of STOPP/START criteria can reduce both the incidence of ADRs and medication costs in older, hospitalised patients [16, 17]. Application of the STOPP/START version 2 criteria into a structured medication review process is defined as the Systematic Tool to Reduce Inappropriate Prescribing (STRIP) [18].

More recently, the European Commission and Swiss Government-funded OPERAM (OPtimising thERapy to prevent Avoidable hospital admissions in the Multi-morbid elderly) project was established based on the use of the STRIP medication review. The STRIP process encompasses the use of a customised software-based tool known as the STRIP Assistant (STRIPA), which was developed to support healthcare professionals to perform the STRIP medication review process. The STRIPA process then generates a report with prescribing recommendations addressing potentially inappropriate prescribing (PIP) or potential prescribing omissions (PPOs) [19]. STRIPA consists of four main components, i.e. functional architecture, user interface, decision rule engine, and semantic interoperability [20]. For the purpose of the multi-centre OPERAM trial, the STRIPA software was translated into four languages; English, German, French and Dutch.

Integration of STOPP/START criteria into a stand-alone web-based clinical decision support system (CDSS) could improve the detection of inappropriate prescribing. A recent review has demonstrated that computerised interventions can significantly decrease PIP in hospitalised older adults, although the authors highlight that larger scale multinational RCTs are needed to support this contention [21]. Interestingly, other studies that investigated the benefits of medication review software based on clinical tools such as STOPP/START confirm the high identification rate of PIP, but address the fact that this can result in less clinically relevant recommendations being made [22]. Furthermore, it has been shown that the majority of DRPs identified during medication review may not be associated with the STOPP/START criteria [23]. Taken together, these results suggest that the application of STOPP/START alone does not adequately detect all drug-related errors and that consequently a more complex intervention is necessary to optimise the medication review process. Therefore, a structured assessment, including a patient interview that identifies health and medication issues, combined with a medication review facilitated by a CDSS and evaluated by trained healthcare professionals, could potentially identify the most relevant drug-related problems.

**Fig. 1:**
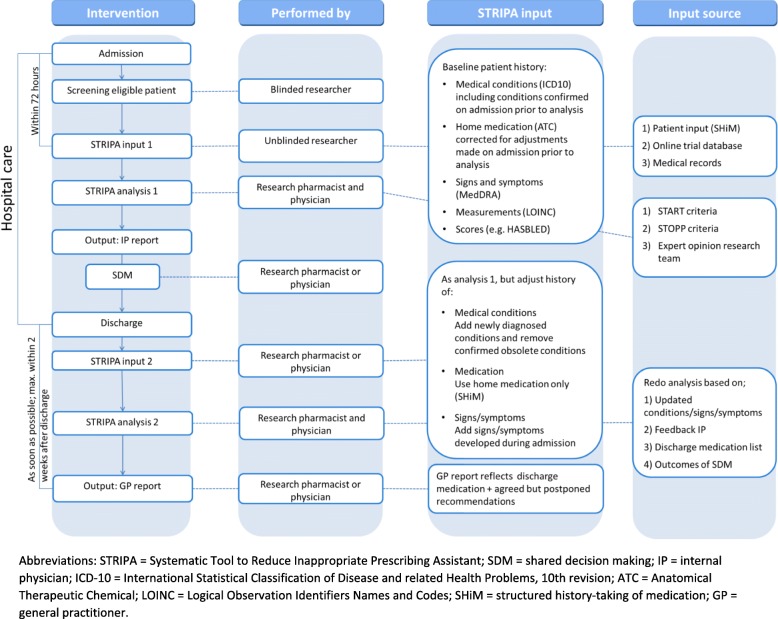
Flowchart of STRIP and STRIPA intervention process. Abbreviations: STRIPA = Systematic Tool to Reduce Inappropriate Prescribing Assistant; SDM = shared decision making; IP = internal physician; ICD-10 = International Statistical Classification of Disease and related Health Problems, 10th revision; ATC = Anatomical Therapeutic Chemical; LOINC = Logical Observation Identifiers Names and Codes; SHiM = structured history-taking of medication; GP = general practitioner

The aim of the OPERAM study is to assess the impact of a structured drug review utilising the STRIP method, including STRIPA software, on the quality of pharmacotherapy and whether such optimisation of pharmacotherapy in older people can reduce the number of drug-related hospital admissions in older patients with multi-morbidity and polypharmacy hospitalised previously (i.e. at enrolment into OPERAM) [24].. The trial protocol has been described elsewhere [25]; the aim of this report is to describe the structured, multi-component intervention and compare it with the approach in the comparator arm (see Fig. 1. Flowchart of STRIP and STRIPA intervention process). This protocol has been written in line with Standard Protocol Items: Recommendations for Interventional Trials (SPIRIT) recommendations (see **Additional File 1**).

## Methods/design

### Intervention arm

#### The STRIP intervention as in OPERAM

- *Step 1: Structured History-taking of Medication (SHiM)*

In order to optimise patients’ pharmacotherapy during their hospital stay, their medication lists have to be as accurate as possible at the point of arrival. Several studies have shown that older patients’ medication lists on admission to hospital significantly differ from what they actually take at home [26–29]. These differences can be of clinical significance, causing adverse drug events (ADEs) or patient harm [30, 31] and older patients are particularly at risk from these events [32]. Medicines reconciliation as an intervention has repeatedly been shown to reduce medication discrepancies and to improve the accuracy of medication lists [26, 29], although there is no clear consensus on the most accurate method of carrying out medicines’ reconciliation. Different sources for obtaining information on medication history include letters from referring physicians, community pharmacy dispensing lists and patients’ own medications, although none of these methods is completely accurate when taken in isolation and the use of several sources is recommended [31]. To address this problem, the Structured History-taking of Medication (SHiM) was devised by Spee and colleagues [33] who developed a 21-item questionnaire that can be used to fully interrogate a patient’s current medication use (including non-prescription medications), patient’s attitudes and beliefs towards their own medication regime, any perceived barriers to medication use as well as any known medication allergies or intolerances [28]. Application of the SHiM has been shown to successfully detect discrepancies in medication lists in up to 92% of patients being admitted to hospital, reducing potential patient harm as a result of addressing these errors [28, 34].

In OPERAM, a SHiM assessment is conducted for all intervention patients, either with the patients themselves or their next-of-kin in the case of patients with cognitive impairment, typically between 24 and 72 h after inclusion in the trial. It is completed by a trained researcher (pharmacist, physician or nurse) and is performed separately to the routine clinical history-taking which is completed on admission by a member of the attending medical team. In OPERAM, a modified version of the SHiM is used, which has removed the final 7 questions from previously described versions [28] (see Table 1. Questions in the modified SHiM used in the OPERAM trial). In addition to the SHiM, at least one other source is consulted. Preferably, a complete medication dispensing list is obtained from the community pharmacy and/or the general practitioner (GP), or if not available, a list of medications on admission is taken from the patient’s medical records or from the primary care physician’s referral letter.

- *Step 2: Clinical Decision Support System with integrated STOPP/START (STRIPA)*

**Table 1:** Questions in the modified SHiM used in the OPERAM trial

***Questions on individual drug level***	
1. Are you using this drug as prescribed? (dosage, dose frequency and dosage form)	
2. If not, what is the reason for deviating (from dosage, frequency or form) or not taking the drug at all?	
3. Are you experiencing any side-effects from taking this drug?	
***Questions on a general level***	
4. Are you using any other prescription drugs that are not mentioned on this list?	
5. Are you using non-prescription drugs?	
6. Are you using homeopathic drugs or herbal medicines?	
7. Are you using drugs that belong to family members or friends?	
8. Are you using any ‘as needed’ drugs?	
9. Are you using drugs that are no longer prescribed?	
10. Do you have any drug allergies?	
11. Do you have any drug intolerances?	

The pharmaceutical analysis within the OPERAM trial is carried out by a trained research physician and a trained research pharmacist in mutually supportive roles assisted by the STRIPA software. STOPP/START criteria (version 2) were converted into clinical rules though an extensive, multi-disciplinary process, and these rules were then incorporated into the stand-alone CDSS to assist clinicians in detection of PIP and PPOs. However, suggestions can also be manually entered based on expert opinion by the trained research physician or pharmacist. Within STRIPA, the patient demographic data are entered anonymously, and baseline data including details of age, gender and race are recorded. Race is entered as either black or non-black for the sole purpose of calculating the estimated Glomerular Filtration Rate (eGFR) using the Chronic Kidney Disease Epidemiology Collaboration (CKD-EPI) formula [35, 36].

The patient clinical data are then entered as medical conditions using the International Statistical Classification of Disease and related Health Problems, 10th revision (ICD-10) codes, current medications as Anatomical Therapeutic Chemical (ATC; level 5) codes and measurements such as blood pressure, bone mineral density and laboratory values using Logical Observation Identifiers Names and Codes (LOINC) codes. The different steps taken during data entry and analysis will now be described in greater detail.

### Data entry

After entering the baseline patient characteristics, the patient’s medical data are entered in five sequential steps:

1. All relevant medical conditions (either chronic or acute) are entered using ICD-10 codes. Surgical interventions not requiring (current) medical treatment are not considered for data input. Coronary artery stent deployment, for example, is entered as this treatment requires antiplatelet therapy for 6–12 months. For some medical conditions, the date of onset is important and this can also be entered during this step.
2. All current medications are entered (including those upon admission) at ATC-5 level (generic drug names), including frequency and route of administration. This may differ from the patient’s home medication. Additionally, drugs with a long-term indication that have been withheld upon admission due to the specific nature of the patient’s presenting illness are included, as their re-initiation after hospitalization is likely.
3. All patient-reported signs and symptoms are entered. They are either elicited from the patient during SHiM or found in the medical records or in the laboratory results. A predefined list of signs and symptoms present in START and STOPP criteria in the form of checkboxes is available in STRIPA, and includes for example constipation, dizziness, blurred vision and ankle oedema, among others. Other signs or symptoms can be entered manually and then selected from the Medical Dictionary for Regulatory Activities (MedDRA) database, a medical dictionary developed by the International Council for Harmonization of Technical Requirements for Pharmaceuticals for Human Use (ICH), integrated with STRIPA.
4. All available vital and laboratory measurements are reviewed. However, only those parameters present within one or more of the STOPP and START criteria are available within STRIPA. These can either be entered manually or selected from the predefined list of parameters present.
5. The final step in the data entry process comprises different measurements, specifically the HAS-BLED score [37], clinical parameters such as urea and electrolyte values, heart rate and blood pressure, patient height and weight as well as the pneumococcal and influenza vaccination status. Additionally, allergies and ADRs can be entered here as plain text.

### STRIPA analysis

**Fig. 2:**
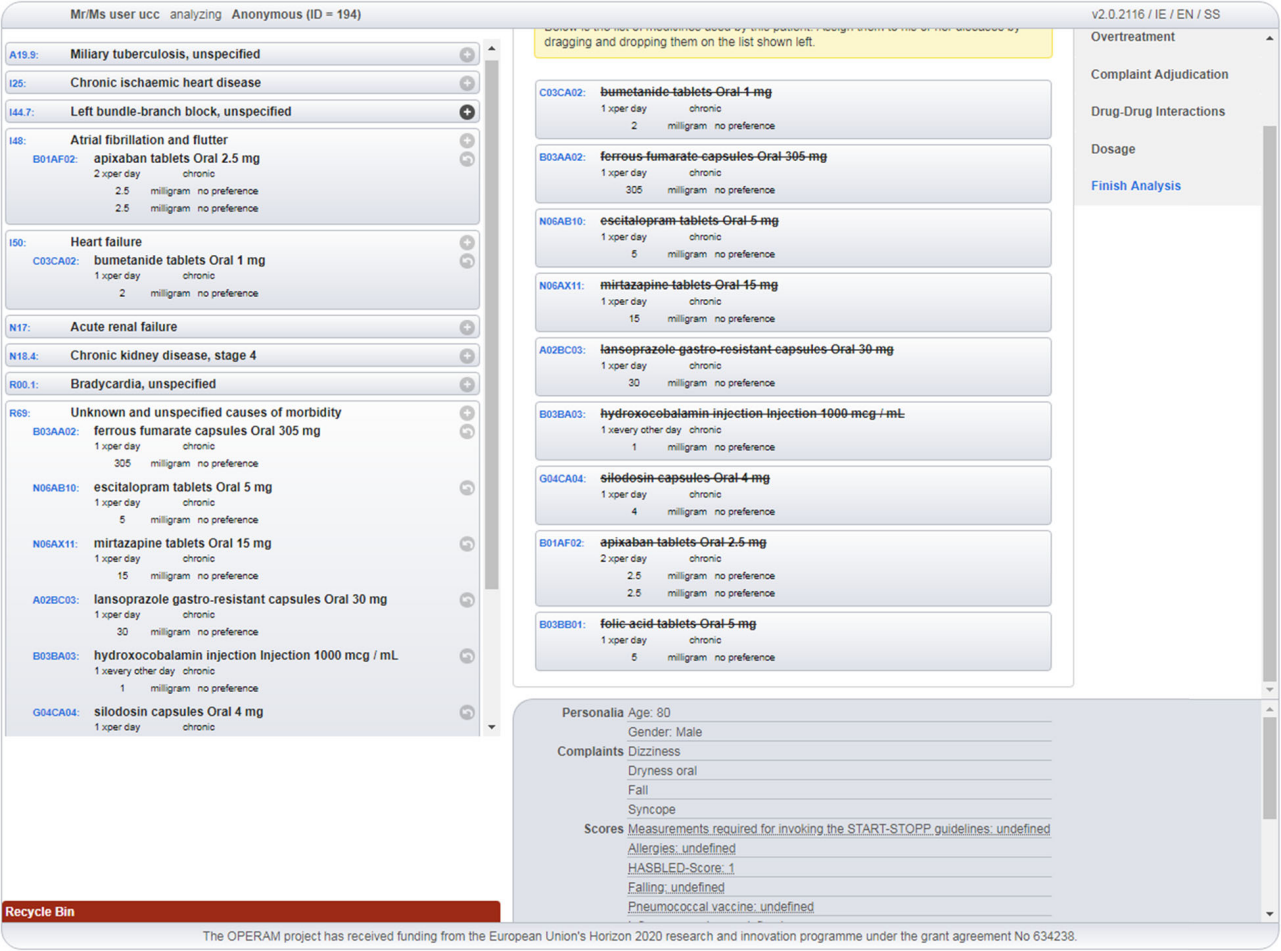
Screenshot of STRIPA process during which medications are assigned to relevant medical conditions

**Fig. 3:**
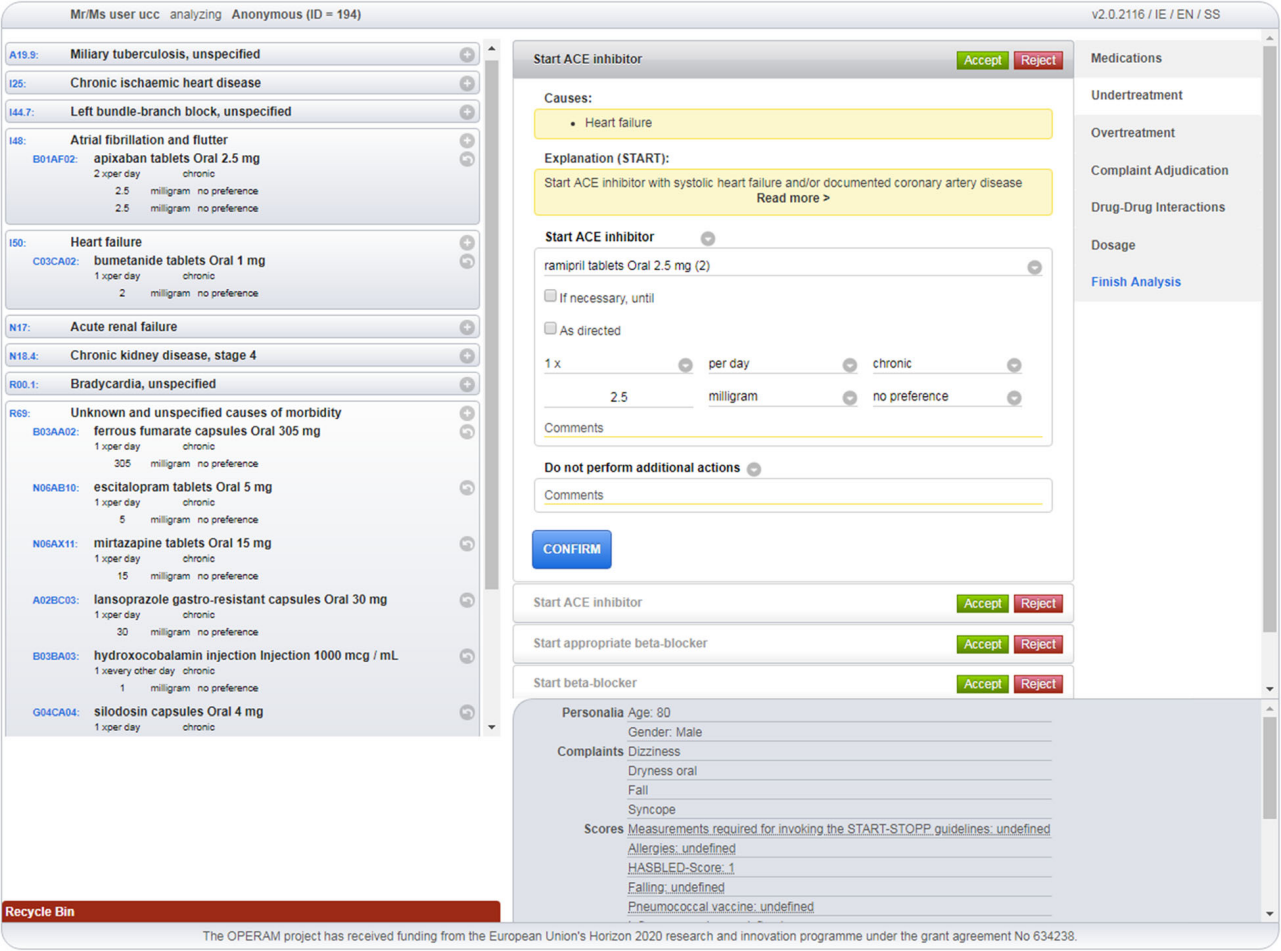
A screenshot of triggered START criteria

The pharmaceutical analysis consists of six steps, according to the Prescribing Optimization Method [38], at the end of which a report with prescribing recommendations is generated. These steps are as follows:

1. Assignment of medication to the recorded diagnoses: the STRIPA user assigns all the entered *medications* to the present ICD-10 codes representing the patient’s medical conditions (see Fig. 2**.** Screenshot of STRIPA process during which medications are assigned to relevant medical conditions). This can be achieved by ‘*dragging’* the medications by screen cursor on the ‘*right side’* of the screen to the corresponding indicated medical condition on the *‘left side’* of the screen. Where no appropriate indication for a medication is present, this medication can be assigned to ICD-10 code ‘*R69- unknown and unspecified causes of morbidity*’, i.e. a so-called ‘dummy condition’.
2. Screening for under-treatment: during this step, the entered medications and medical conditions are checked for *under-treatment* according to START criteria (see Fig. 3**.** A screenshot of triggered START criteria). All medications assigned to a medical condition are evaluated, regardless of the specific medical condition they were assigned to. For instance, where an ACE inhibitor is assigned to hypertension instead of heart failure, START rule A6 (“*Angiotensin Converting Enzyme (ACE) inhibitor with systolic heart failure and/or documented coronary artery disease*”) will not be triggered as the ACE inhibitor is already present in the medication list. The intervention team will evaluate all generated START rules on their appropriateness for a specific patient by either accepting or rejecting the advice. In the event of a rejected recommendation, the reasons for rejection are not recorded within the STRIPA software. When a START recommendation is accepted, the user can choose any medication on an ATC-5 level, including preferred dose, within the advised class from a drop-down menu. This drug is then automatically assigned to the medical condition triggering the rule. When more than one criterion is triggered advising the same drug (or drug class), the best matching criterion is chosen by the intervention team and the others are then automatically disabled. At the end of this step, the updated medication list is evaluated for potential under-treatment not highlighted in START criteria, but considered relevant according to the STRIPA software user. In such cases, these drugs can be manually added to the designated medical condition and will appear on the final advice report as ‘*expert opinion’* instead of triggered by START criteria.
3. Screening for over-treatment: this step involves evaluation of *over-treatment* according to STOPP criteria. All medications including those initiated in the prior step are evaluated based on the medical conditions and known biomedical parameters and symptoms or complaints. During this step, the newly initiated medications, including START criteria-based recommendations accepted during the previous step, could also appear as STOPP recommendations. For example, in the previous step an ACE inhibitor was started according to START rule A6. However, due to the presence of hyperkalaemia, STOPP rule B11 (“*ACE Inhibitors or Angiotensin Receptor Blockers in patients with hyperkalaemia*”) would then be triggered. The user decides whether these STOPP recommendations are relevant to the patient under review. If a recommendation is followed, the medication in question will then be removed from the recommended medications list. They will appear on the final report as *‘medication advised to be stopped’*. All medications that could not be assigned to an appropriate medical condition and have therefore been allocated the ICD-10 code ‘*R69’* are considered potential overtreatment. Moreover, the STOPP criteria addressing impaired renal function and combinations with certain medications (e.g. digoxin and eGFR < 30 ml/min) will be triggered here, based on either entered eGFR values or an ICD-10 diagnosis of renal insufficiency. In addition to stopping medications, the user could also decide to recommend a dose adjustment (both manually and based on STOPP criteria).
4. Medication-Disease Interactions (ADEs): this step encompasses the adjudication of clinical signs or symptoms entered which are based on the predefined list of symptoms and signs that may be attributable to medications or medical conditions. The software user, based on expert opinion, can assign symptoms and signs manually to medications and a drop-down menu with three possible actions appears: (A) The symptom/sign can be registered as ‘*side effect’* of the concerning medication; (B) The medication can be either maintained, stopped or adjusted; (C) Adaptations to other drugs can be made including stopping, adjusting or starting new drugs. All assigned symptoms and signs will appear on the report linked to their possible causative medication.
5. Medication-Medication Interactions: during the fifth step, the medication list will be checked for drug-drug interactions based upon the incorporated or local interaction database (dependent on licensing) within the software. If an interaction is identified, the user can again choose to act upon or ignore the prompt. An explanation about the interaction is present to assist the software user in this decision process. When a drug-drug interaction is addressed, the software user must decide which medication to maintain, stop or adjust. Also, other drugs from the medication list can be adapted here and a new medication can be initiated, for instance to replace one of the interacting medications.
6. Dosage: the final step consists of dose adjustment recommendations based on the Dutch KNMP Kennisbank® database and the patient’s calculated eGFR. When a recommendation is acted upon, the software user can choose to maintain, stop or adjust the concerned medication and/or take other actions including adjustment of other medications in the list or starting a new medication.

**Fig. 4:**
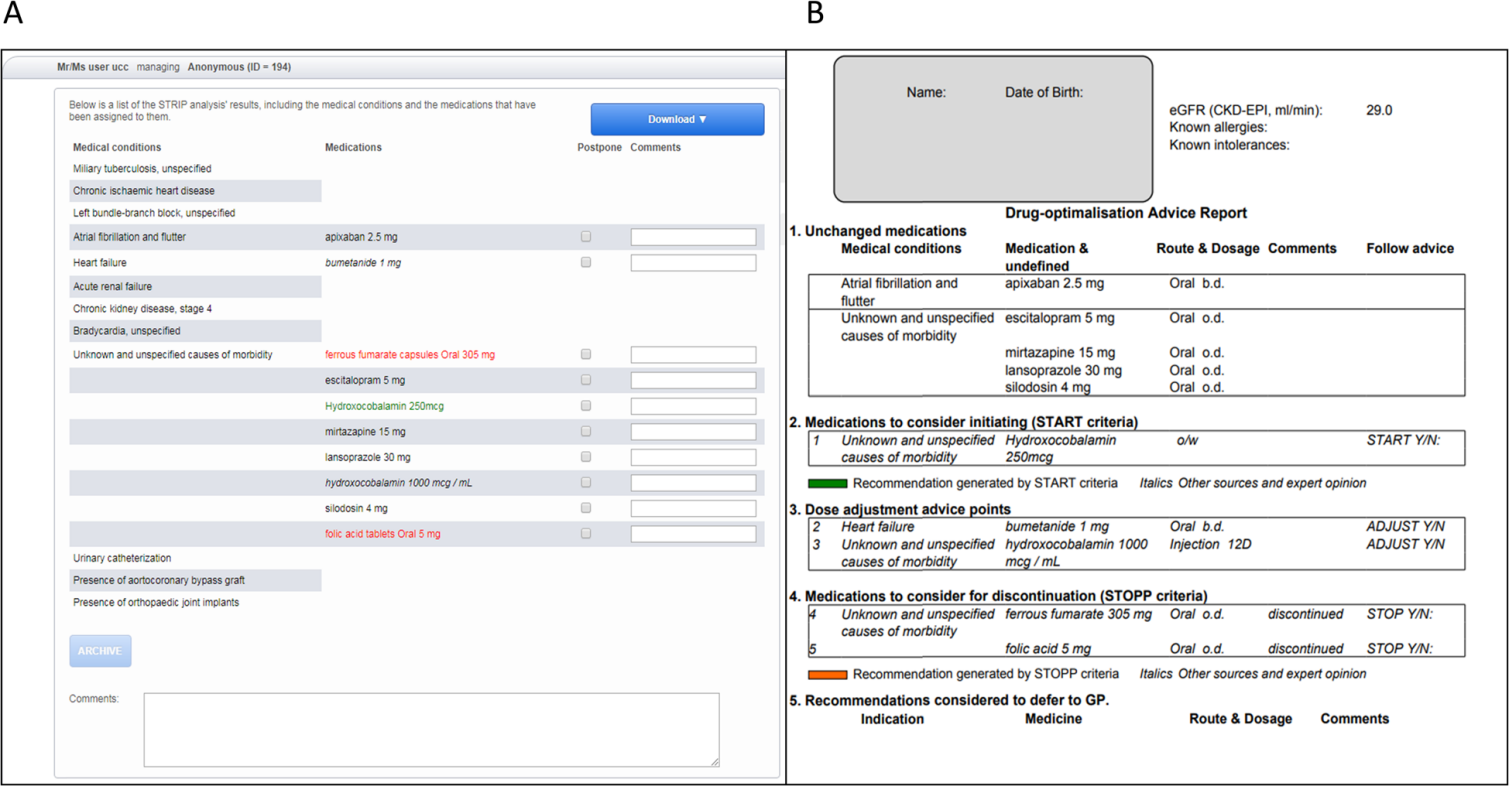
The internal physician report: (**a**) final screen in the STRIPA process, and (**b**) completed report

After completing the steps above, the analysis is finalized. All choices made are then saved within the STRIPA system and tracked in the background. However, the different steps of the analysis can be revisited at all times, if necessary. When the analysis is considered complete, an overview of all the adaptations to the medication list can be viewed in the ‘*advice tab’*. Here, all suggested medications to be discontinued are shown in red, newly started medications are in green and manually adjusted medications appear in italics*.* The medications are still linked to the corresponding medical condition and will appear correspondingly on the report. In the advice tab, the user can manually adapt the plain text of both medical conditions and medications to enhance the final report presented to the patient’s prescribing (internal) physician (see Fig. 4a**.** The internal physician report: (A) final screen in the STRIPA process, and (B) completed report). This will not affect the underlying ATC and ICD-10 codes saved in the STRIPA track. Furthermore, comments on the recommendations (other than explanations of STOPP and START criteria which will appear on the report regardless) can be added by the user according to each proposed medication change in order to convince the prescribing physician to follow the advice or to emphasize the importance of the recommendation. Moreover, recommendations can be deferred to the patient’s primary care physician when they are not deemed appropriate to the current acute clinical situation. Lastly, a general comment box exists where the software users can enter extra information or considerations regarding the recommendation or general points of attention relevant to this patient. After all adaptations are made, the report known as the ‘internal physician report’ (see Fig. 4b**.** The internal physician report: (A) final screen in the STRIPA process, and (B) completed report) can be downloaded and printed for discussion with the prescribing hospital physician.

- *Step 3: Communication and discussion of the STRIPA report with the prescribing physician*

After the first analysis has been conducted and the prescribing physician report is complete, the research pharmacist and research physician contact the prescribing physician and discuss the implementation of the STRIPA-generated recommendations. The objective is to incorporate the prescribing recommendations with the insight that the prescribing physician can provide with regards to the overall functional capacity of the patient to reach a consensus about the recommendations that should be implemented to prevent both ADRs during the hospital stay, and later drug-related readmissions (i.e. the primary endpoint of the OPERAM trial).

- *Step 4. Shared-decision making with the patient*

Subsequently, once consensus has been reached between the researchers and the prescribing physician, the process of shared decision-making (SDM) can take place if the prescribing physician has identified preference-sensitive decisions with regard to stopping, starting, continuing or selecting medications for discussion with the patient. SDM has been defined as “*an approach where healthcare professionals and patients share the best available evidence when faced with making decisions regarding healthcare, and where patients are supported to consider options to achieve informed preferences”* [39]. This process addresses patients’ autonomy and promotes patient engagement [39], and it has repeatedly been shown to play an integral role in a successful de-prescribing of harmful drugs [40–42].

The model for SDM has previously been described elsewhere [43]. Briefly, it is centred around 4 main principles i.e. ‘choice talk’, ‘option talk’, ‘preference talk’ and ‘decision talk’ [43]. All patients, in particular patients with cognitive impairment, should be facilitated to have another relevant person (e.g. close family member) present when making any decisions in the SDM process. Collectively, the research team and the patient agree on definitive medication changes to be made and then proceed to develop a pharmaceutical care plan. Changes after the SDM process are communicated to the prescribing physician, and in some cases, the SDM can be deferred to the patient’s GP; if so, this is documented on the GP information letter, as will be discussed in the next section.

- *Step 5: Discharge and the GP information report*

Once recommendations are agreed between the research team, the prescribing physician and the patient, the changes to the patient’s medications are entered into STRIPA and a report known as the *“GP report”* is generated. Where the prescribing physician has accepted STRIPA recommendations, these recommendations are included in the GP report. Where the prescribing physician has made changes unrelated to STRIPA, these changes are entered manually. In cases where SDM is deferred to the GP, instructions for the GP are written by either the research physician or research pharmacist in the section of the GP report entitled “*recommendations not yet applied during hospitalization*”. The GP report should then be identical to the patient’s discharge prescription, and is mailed to the GP after the patient is discharged from hospital.

### Control arm and SHAM intervention

Patients in the control group receive usual care, with the potential of a medication review by the prescribing physician in accordance with usual pharmaceutical care. Patients from both groups complete the 8-item Moriskey Medication Adherence Scale questionnaire (MMAS-8) [44] with a trained member of the intervention team. This is to prevent potential unblinding in the event of unblinded team members approaching patients when attending patients’ wards.

### Device deficiency

Due to a software tool being used in this trial, there is the potential for a so-called device deficiency, defined by the European Medical Device Vigilance System (MEDDEV) 2.7/3 [45] as an “*Inadequacy of a medical device related to its identity, quality, durability, reliability, safety or performance. This may include malfunctions, use error, or inadequacy in the information supplied by the manufacture.*” All technical problems with the STRIPA system are reported, using the designated STRIPA feedback form, within 24 h to the software developers, who then assess whether the problem in question is a possible device deficiency. They will then report back within 72 h to the clinical site in question with details of the investigation of the issue and determine any actions to be taken. If corrective actions are required at all sites, all co-Principal Investigators (PIs) including the co-ordinating PI are informed within another 48 h.

### Safety section

The STRIPA software provides general recommendations and is not intended to impose firm decisions. It does not replace decision-making and clinical judgements made by physicians and pharmacists and this is explicitly stated in the disclaimer on the printed reports. It is expected that prescription recommendations made by the STRIPA system that turn out to be inappropriate for an individual patient are detected by a pharmacist or physician conducting the intervention and addressed appropriately to safeguard patients’ welfare. The prescribing physicians remain responsible for all final medical decisions concerning their patients.

## Discussion

ADRs, which are particularly likely to occur during acute hospital admission, cause significant morbidity in older patients and contribute to increased healthcare costs [45]. ADRs are common in older multi-morbid patients and often lead to acute hospitalization despite reports that approximately 50% of these drug-related admissions (DRA) are likely to be preventable [7, 46]. Growing evidence indicates that optimising pharmacotherapy, through various interventional designs, mitigates inappropriate prescribing as well as the incidence of ADRs and associated costs in this high-risk patient population [11, 15, 16]. Although there is insufficient data to support the use of a single validated intervention, a recent review highlighted the value of several methods including close liaison between physicians and clinical pharmacists as well as the use of implicit and explicit prescribing criteria such as STOPP/START [11]. A particular strength of the OPERAM trial is its novelty, i.e. it is one of the first computerised interventions designed to incorporate a structured medication review to look at potentially inappropriate prescribing and potential prescribing omissions in older hospitalised patients, and assesses whether it reduces drug-related hospital admissions. It also recognises the importance of the identification of patient-reported clinical signs and symptoms that may be related to PIP. Moreover, it relies on multi-disciplinary input and collaboration between physicians and pharmacists and clear communication of prescribing information with GPs, which will likely increase the impact of prescribing recommendations on patient care. Finally, the SDM process allows for greater emphasis to be placed on a patient-centred approach, encouraging patient engagement with their own healthcare. The integration of multiple interventions that have demonstrated benefit is anticipated to have a synergistic effect on pharmacotherapy quality. The study can also demonstrate the feasibility of a multi-component intervention in a hospital environment. A key strength of the OPERAM trial will be its demonstration of feasibility in differing healthcare environments of the EU and non-EU countries. The OPERAM trial will also analyze the intervention from a health economics perspective and will allow for the determination of the benefit that the intervention can provide to society in general through a reduction in healthcare expenditure. Recruitment for the OPERAM trial began in December 2016 and finished in October 2018. Trial follow-up will be completed in October 2019 and trial results are expected in the first quarter of 2020.

## Data Availability

Data will be deposited in the Bern Open Repository and Information System (BORIS) (www.boris.unibe.ch). BORIS allows searching and is indexed by search engines. All items are stored with a unique Digital Object Identifier (DOI) that can be referenced in respective publication. The whole study database will be in csv format, and will include README files, metadata, information about the performed processing and analytical steps, variable definitions, and references to vocabularies used to help secondary users to understand and reuse the data. Data will only be shared upon request. Data use proposals will be evaluated by the OPERAM publication committee. The data is owned by the sponsor-investigators. In case of data sharing, a data-sharing agreement between the external party and the sponsor-investigator will need to be agreed on and signed.

## Abbreviations

ACE: Angiotensin-converting enzyme
ADE: Adverse drug event
ADR: Adverse drug reaction
ATC: Anatomical therapeutic chemical
CDSS: Clinical decision support systems
CKD-EPI: Chronic kidney disease epidemiology collaboration
DRA: Drug-related admissions
DRP: Drug-related problem
eGFR: Estimated glomerular filtration rate
GP: General practitioner
ICD-10: International Statistical Classification of Disease and related Health Problems, 10th revision
ICH: International Council for Harmonization of Technical Requirements for Pharmaceuticals for Human Use
KNMP: Koninklijke Nederlandse Maatschappij ter bevordering der Pharmacie (Royal Dutch Pharmacists Association)
LOINC: Logical observation identifiers names and codes
MEDDEV: European Medical Device Vigilance System
MedDRA: Medical Dictionary for Regulatory Activities
MMAS-8: 8-item Moriskey Medication Adherence Scale questionnaire
OPERAM: Optimising thERapy to prevent Avoidable hospital admission in the Multi-morbid elderly
PIP: Potentially inappropriate prescribing
PPO: Potential prescribing omissions
QoL: Quality of life
RCT: Randomised controlled trials
SDM: Shared decision making
SHiM: Structured history taking of medication
START: Screening tool to alert to right treatment
STOPP: Screening tool of older persons’ prescriptions
STRIP: Systemic tool to reduce inappropriate prescribing
STRIPA: STRIP Assistant
